# Temperature Activated Dimensionality Crossover in the Nucleation of Quantum Dots by Droplet Epitaxy on GaAs(111)A Vicinal Substrates

**DOI:** 10.1038/s41598-019-51161-5

**Published:** 2019-10-10

**Authors:** Artur Tuktamyshev, Alexey Fedorov, Sergio Bietti, Shiro Tsukamoto, Stefano Sanguinetti

**Affiliations:** 1 L–NESS and Department of Material Science, University of Milano-Bicocca, Milano, 20125 Italy; 2 L-NESS and CNR–IFN, Como, 22100 Italy

**Keywords:** Quantum dots, Quantum dots

## Abstract

A temperature activated crossover between two nucleation regimes is observed in the behavior of Ga droplet nucleation on vicinal GaAs(111)A substrates with a miscut of 2° towards $(\bar{1}\bar{1}2)$. At low temperature (<400 °C) the droplet density dependence on temperature and flux is compatible with droplet nucleation by two-dimensional diffusion. Increasing the temperature, a different regime is observed, whose scaling behavior is compatible with a reduction of the dimensionality of the nucleation regime from two to one dimension. We attribute such behavior to a presence of finite width terraces and a sizeable Ehrlich-Schwöbel barrier at the terrace edge, which hinders adatom diffusion in the direction perpendicular to the steps.

## Introduction

The understanding and the control of the phenomena related to the self-assembly of compound semiconductor epitaxial quantum dots (QDs) is an extremely interesting field of research as it blends fundamental physics and device application aspects. As a matter of fact, epitaxial QDs have found application in a multitude of photonic devices such as QD lasers, photodetectors, single, and entangled photon emitters^1–4^. Each application drives the system toward different size, shape and density as the electronic properties of the QDs strictly depends on these physical quantities.

Droplet epitaxy (DE) is a flexible growth method, performed in a Molecular Beam Epitaxy (MBE) environment, which allows to self-assembly strain free, shape engineered QDs^5^. DE-QD formation process consists of two stages. First, liquid metal droplets are grown in a Volmer-Weber-like mode, followed by crystallization and transformation into semiconductor QDs by annealing in As atmosphere^6^. DE, permits the self-assembly on a large variety of substrates, including (111) exact and vicinal surfaces^5^_._ Such surface orientation, owing to the intrinsic C_3v_ symmetry, is relevant for the novel application of QDs in quantum photonics as it permits to self-assembly QDs with a negligible fine-structure splitting (FSS) of the excitonic emission^7^ and thus, in turn, entangled photon emission. Particular relevance have (111)A vicinal surfaces, which, due to the presence of steps, permit the growth of flat surfaces and the implementation of Bragg reflectors into the sample structure.

Fundamental DE-QD properties, volume and density, are then controlled by the droplet characteristics. Understanding the metal droplet nucleation process on (111)A and vicinal surfaces in QD-DE self-assembly is, therefore, a fundamental step for improving presented entangled photon emitters. As shown by Venables *et al*.^8,9^, the island density *N* dependence on the deposition rate *F* and temperature *T* gives access to a material parameter, the size of the critical nucleus, *i* (the number of atoms that are part of the largest unstable cluster) which is a fundamental parameter for the description of the physical phenomena occurring during the deposition stage. The way *N* depends on *i* is determined by the actual physically relevant nucleation regime occurring during the growth^10^.

Here we show the presence, on GaAs(111)A substrates with a miscut of 2° towards $(\bar{1}\bar{1}2)$, of a temperature activated crossover in the dimensionality of the scaling parameters that controls the dependence of droplet density *N* on *F* and *T* from two to one dimension. We correlate such crossover to the presence of the temperature activated onset of a restriction in the adatom diffusion related to the presence of regular and finite width terraces and steps. The latter, due to the associated Ehrlich-Schwoebel (ES) barrier^11^, induce a strong spatial anisotropy in the adatom diffusivity at the origin of the dimensionality change.

## Experimental Methods

Samples were grown with an molecular beam epitaxy (MBE) system, equipped with a valved cell for As_4_ supply, on a semi-insulating GaAs(111)A substrates with a miscut of 2° towards $(\bar{1}\bar{1}2)$. Substrates temperatures were measured by thermocouple situated between the substrate heater and the sample and by infrared pyrometer. We observed a good reproducibility of the temperature (580 °C) for native oxide desorption and appearance of (2 × 2) reconstruction^12^. Every growth experiment was *in-situ* monitored by reflection high energy electron diffraction (RHEED). The *ex-situ* morphological characterization of the samples was performed by an atomic force microscope (AFM) in tapping mode, using tips capable of a resolution of about 2 nm.

After oxide desorption at 590 °C, a 130 nm GaAs buffer layer was deposited at growth temperature of 520 °C, in order to obtain a smooth surface. The growth rate was 0.5 ML/s (here and below 1 ML is defined as 6.26 × 10^14^ atoms/cm^2^, which is the site-number density of the unreconstructed GaAs(001) surface). The beam equivalent pressure (BEP) of As_4_ flux was set at 2 × 10^−5^ Torr. During the growth of the buffer layer deposition, only (2 × 2) reconstruction is observed. After the growth of buffer layer, the surface was smoothed out at the same temperature and As_4_ BEP of 8.7 × 10^−6^ Torr for 5 minutes. The substrate temperature was then decreased to the droplet deposition temperature varying from 300 to 500 °C and As cell valve was closed in order to deplete the growth chamber from the arsenic molecules. Then 2 ML of Ga were deposited with a rate of 0.01–0.08 ML/s. During the Ga deposition background pressure was below 3 × 10^−9^ Torr. The supply of Ga without As_4_ enabled the appearance of Ga liquid droplets on the surface. The surface reconstruction remained (2 × 2) also during this step. Next, As_4_ flux with BEP of 6.2 × 10^−5^ Torr was supplied at the same temperature for 3 minutes, in order to crystallize Ga droplets into GaAs QDs. A sample D2 was prepared without annealing in As_4_ atmosphere, this leaving liquid the Ga droplets on the surface.

Growth conditions of samples are summarized in Table 1.

**Table 1:** Conditions used for the growth of the samples in this work.

Sample	T, °C	Ga flux, ML/s	*N*, cm^−2^
T1	300	0.01	(4.1 ± 0.5) × 10^10^
T2	350	0.01	(5.6 ± 0.5) × 10^9^
T3	400	0.01	(4.7 ± 0.3) × 10^8^
T4	450	0.01	(3.6 ± 0.1) × 10^8^
T5	500	0.01	(1.7 ± 0.1) × 10^8^
F1	350	0.01	(5.6 ± 0.5) × 10^9^
F2	350	0.04	(2.8 ± 0.4) × 10^10^
F3	350	0.08	(4.2 ± 0.4) × 10^10^
F4	450	0.01	(3.6 ± 0.1) × 10^8^
F5	450	0.04	(5.9 ± 0.4) × 10^8^
F6	450	0.08	(7.9 ± 0.3) × 10^8^
D1	350	0.01	(5.6 ± 0.5) × 10^9^
D2	350	0.01	(7.2 ± 1.1) × 10^9^

The deposition temperature, Ga flux rate during Ga droplet deposition, and the density of GaAs QDs (Ga droplets for sample D2) of samples are indicated.

## Results and Discussion

**Figure 1:**
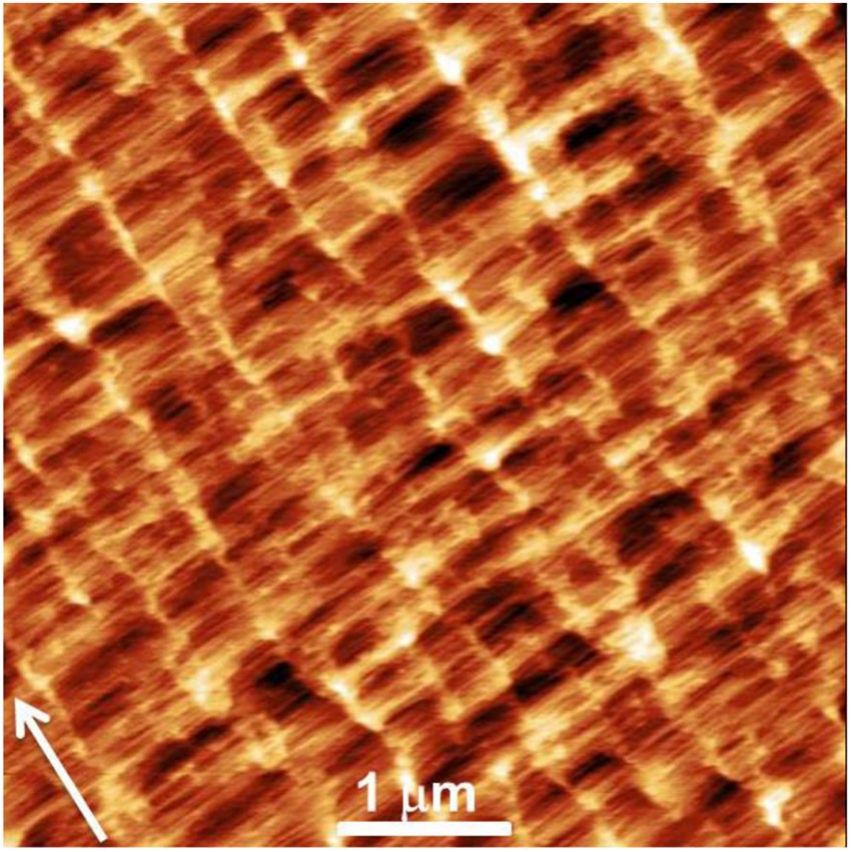
AFM image (5 × 5 μm^2^) of GaAs buffer layer grown on GaAs(111)A with 2° miscut towards $(\bar{1}\bar{1}2)$. Arrow indicates $[\bar{1}\bar{1}2]$ direction perpendicular to steps. The average terrace width is 12.8 nm.

The sample surface topography, measured by AFM, before the metallic Ga deposition is shown in Fig. 1. Via AFM measurements we have determined that the GaAs buffer layer consists of terraces separated by step with height in the 1–3 ML range. The measured average width of the terraces in our conditions is 12.8 nm. (The width of 1 ML terrace is 9.3 nm). The step edges are perpendicular to the $[\bar{1}\bar{1}2]$ direction.

**Figure 2:**
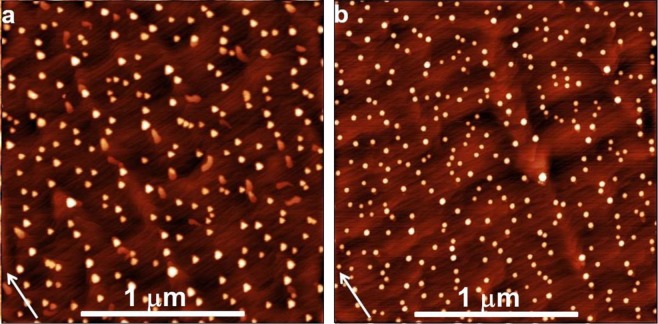
AFM images of (**a**) GaAs QDs (2 × 2 μm^2^, sample D1) and (**b**) Ga droplets (2 × 2 μm^2^, sample D2) grown on GaAs(111)A with 2° miscut towards $(\bar{1}\bar{1}2)$ at 350 °C. Arrows indicate $[\bar{1}\bar{1}2]$ direction perpendicular to steps.

A fundamental starting point for the following discussion is the demonstration that the QD density measured after the annealing in As_4_ atmosphere equals the density of droplets formed during the Ga deposition. This assumption is tested by comparing samples D1 and D2, obtained using the same conditions for the deposition of the Ga droplets. On sample D2 the density of Ga droplets is (7.2 ± 1.1) × 10^9^ cm^−2^, thus in agreement with the density of GaAs QDs measured in sample D1: (5.6 ± 0.5) × 10^9^ cm^−2^. The surface topography of the samples is presented in Fig. 2.

**Figure 3:**
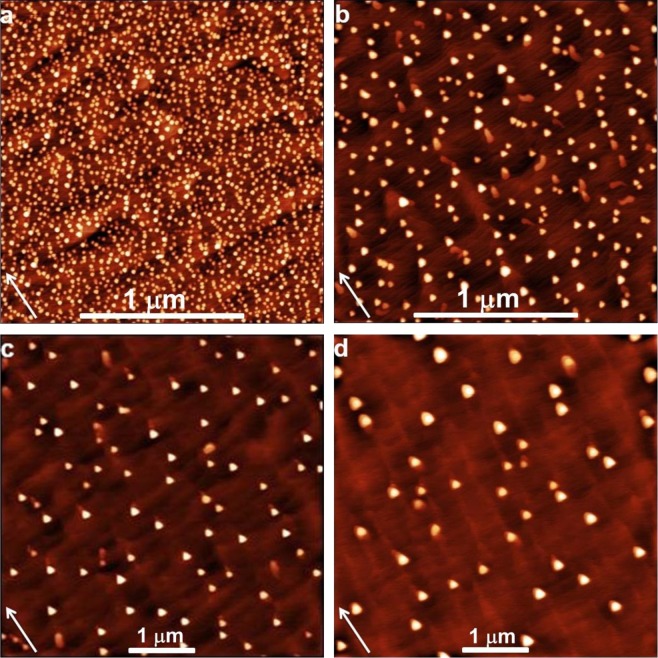
AFM images of GaAs QDs grown on GaAs(111)A with 2° miscut towards $(\bar{1}\bar{1}2)$ at (**a**) 300 °C (2 × 2 μm^2^, sample T1); (**b**) 350 °C (2 × 2 μm^2^, sample T2); (**c**) 450 °C (5 × 5 μm^2^, sample T4); (**d**) 500 °C (5 × 5 μm^2^, sample T5). Arrows indicate $[\bar{1}\bar{1}2]$ direction perpendicular to steps.

The AFM images of the samples T1, T2, T4, T5 are reported in Fig. 3. The droplets are randomly nucleated at the sample surface, with a higher probability at the terrace boundaries. The GaAs(111)A surface is a Ga terminated surface, thus the droplet nucleation does not require to overcome any dose threshold to start, as it has been observed on GaAs(001) substrates^13,14^. The deposition temperature range drives the system in the complete condensation regime, due to the quenching of the Ga desorption^8^. As expected, the droplet density *N* is a function of *T* and *F*. The density of droplet formed by diffusing adatoms on the surface is expected to vary with the power law^8^

1 $$N\propto {(\frac{F}{D})}^{p}$$

where *D* is the adatom diffusivity. The exponent *p* depends on the characteristics of the process of atom aggregation. The *p* parameter is a function of *i* and depends on the actual characteristics of the growth process^10^. Taking into account the exponential dependence of *D*^15^,

2 $$N(F,T)\propto {F}^{p}\cdot \exp ({E}_{a}/{k}_{B}T).$$

Where *E*_*a*_ is a combination of a diffusion activation energy *E*_*d*_ and the nucleation energy of critical cluster *E*_*i*_^8^:

3 $${E}_{a}=p\,\,{E}_{d}+p{E}_{i}/i.$$

Critical cluster size cannot be less than 1, for Ga droplets deposited on singular (001) and (111)A GaAs surfaces the critical cluster size was reported and equals two^16^ and one^17^, respectively. Also for Ga adatoms the nucleation energy *E*_*i*_^16^ is much less than diffusion energy^14^, so we can admit the second term in the Eq. (3) as a small correction, therefore

4 $${E}_{a}\approx p\,\,{E}_{d}$$

**Figure 4:**
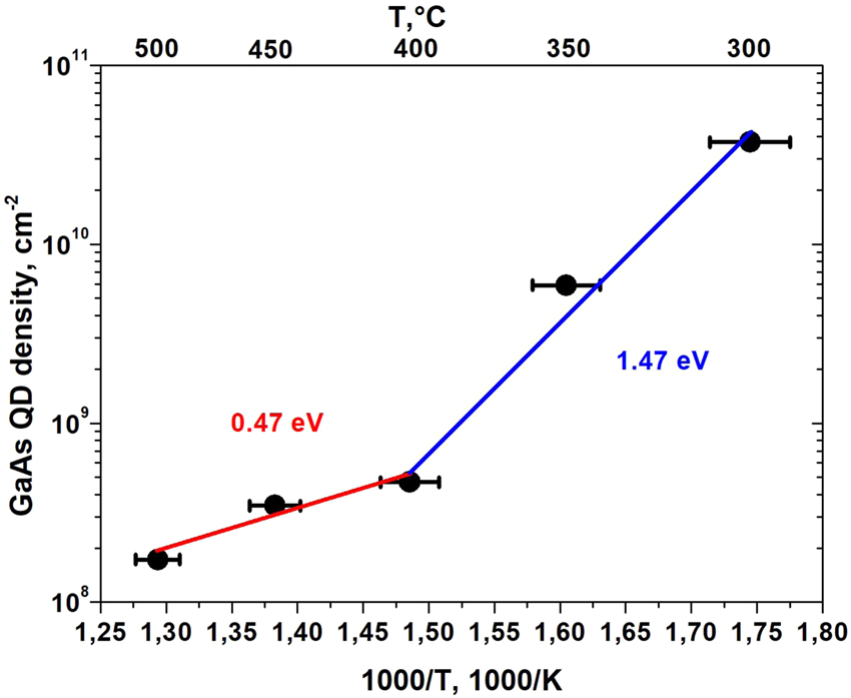
The density of GaAs QDs grown on GaAs(111)A with 2° miscut towards $(\bar{1}\bar{1}2)$ as a function of deposition temperature. 0.47 eV and 1.47 eV are activation energies for two different Ga droplet nucleation regimes.

As reported in Fig. 4, with the increasing deposition temperature, droplet density decreases^18^. A clear bend in the *T* dependence of *N* is observed around 400 °C. The activation energy changes value from *E*_*a*_ = 1.47 ± 0.10 eV at low temperatures to *E*_*a*_ = 0.47 ± 0.03 eV at high temperatures. Here the main error source in the activation energy determination is related to measure of the exact deposition temperature. The presence of two temperature ranges with different activation energies in *N(F,T)* has not been observed on singular (111)A surfaces^18,19^ and, therefore, it is peculiar for vicinal surfaces.

**Figure 5:**
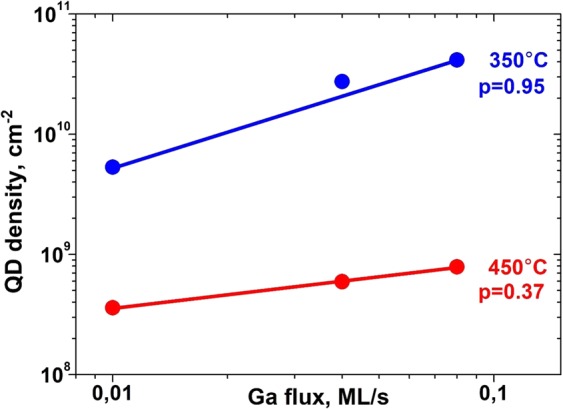
The density of GaAs QDs grown on GaAs(111)A with 2° miscut towards $(\bar{1}\bar{1}2)$ as a function of Ga flux. 0.95 and 0.37 are exponent *p*arameter *p* of density dependence on Ga flux at 350 and 450 °C, respectively.

According to Eq. (2), the change in the Arrhenius plot slope of *N* could be related to a change in the energy *E*_*a*_ due to a temperature modification of the surface diffusion phenomena, or to a variation in the *p* parameter value. *p* can be independently determined via the dependence of *N* on *F* (see Fig. 5). *p* equals 0.95 ± 0.09, and 0.37 ± 0.04 at 350 and 450 °C, respectively, thus showing that a relevant change in the nucleation process is occurring at ≈400 °C.

From the Eq. (4), the diffusion energy *E*_*d*_ is given by the ratio between *E*_*a*_ and *p*. The calculated diffusion energy *E*_*d*_ is 1.54 ± 0.25 eV and 1.27 ± 0.22 eV at 350 and 450 °C, respectively. Therefore, the activation energy for diffusion *E*_*d*_ can be considered as constant, within experimental errors, through the whole measured temperature range. This conclusion is supported by the surface reconstruction, as measured by RHEED, which remains (2 × 2) in the whole temperature range showing that no change in the microscopic diffusion process and thus, in turn, of the diffusion activation energy happens at *T* = 400 °C. The *E*_*d*_ value can be compared with the diffusion energy of Ga adatoms on GaAs(001) surface, estimated by dynamics of Ga droplets^14^ and by indirect *in-situ* RHEED method^20^.

The change with *T* in *E*_*a*_ is therefore related to the change in the *p* parameter. According to ref.^21^, *p* depends on *i* and by the diffusion environment:

5 $${\rm{p}}=\frac{{\rm{i}}}{({\rm{\gamma }}i+{\rm{\gamma }}+1)}$$

Here γ is a parameter which indicated the dimensionality of the diffusion process. For two-dimensional isotropic diffusion, γ = 1. If the adatom diffusion is strongly anisotropic, thus rightfully considered one-dimensional, γ = 2. Any case of restricted adatom diffusion, e.g. in presence of impurities acting as obstacles, is characterized by a parameter γ > 1. A reduction in *p* is therefore related or to a strong decrease in critical island size *i* or to a change in the diffusion dimensionality, which affects the parameter γ. If the observed dependence of *p* on *T* is stemming from a temperature dependence of the critical island size *i*, it would require a change in critical island size, in order to justify the change of *p* of around a factor of 2.5 when temperature crosses *T* = 400 °C, from *i* = 1 at high *T* to *i* ≈ 10 at low *T*, thus too steep and in the wrong direction (lowering the temperature usually stabilizes the nuclei) to be reasonable. In addition, GaAs(111)A-(2 × 2) surface has the Ga-vacancy buckling reconstruction, in which one Ga atom per (2 × 2) unit cell is missing at the outermost Ga layer^22^. It has been shown by scanning tunneling microscopy that initially deposited Ga atoms are consumed to the formation of droplets without filling up the vacancy site^17^. On the other side, a change in the dimensionality of the surface diffusion from two to one dimension may justify the reduction of a factor two of *p* at high *T*. Therefore, it is the utmost importance to independently determine the behavior of adatom diffusivity and the critical nucleus size *i* with deposition temperature on (111)A vicinal surface.

**Figure 6:**
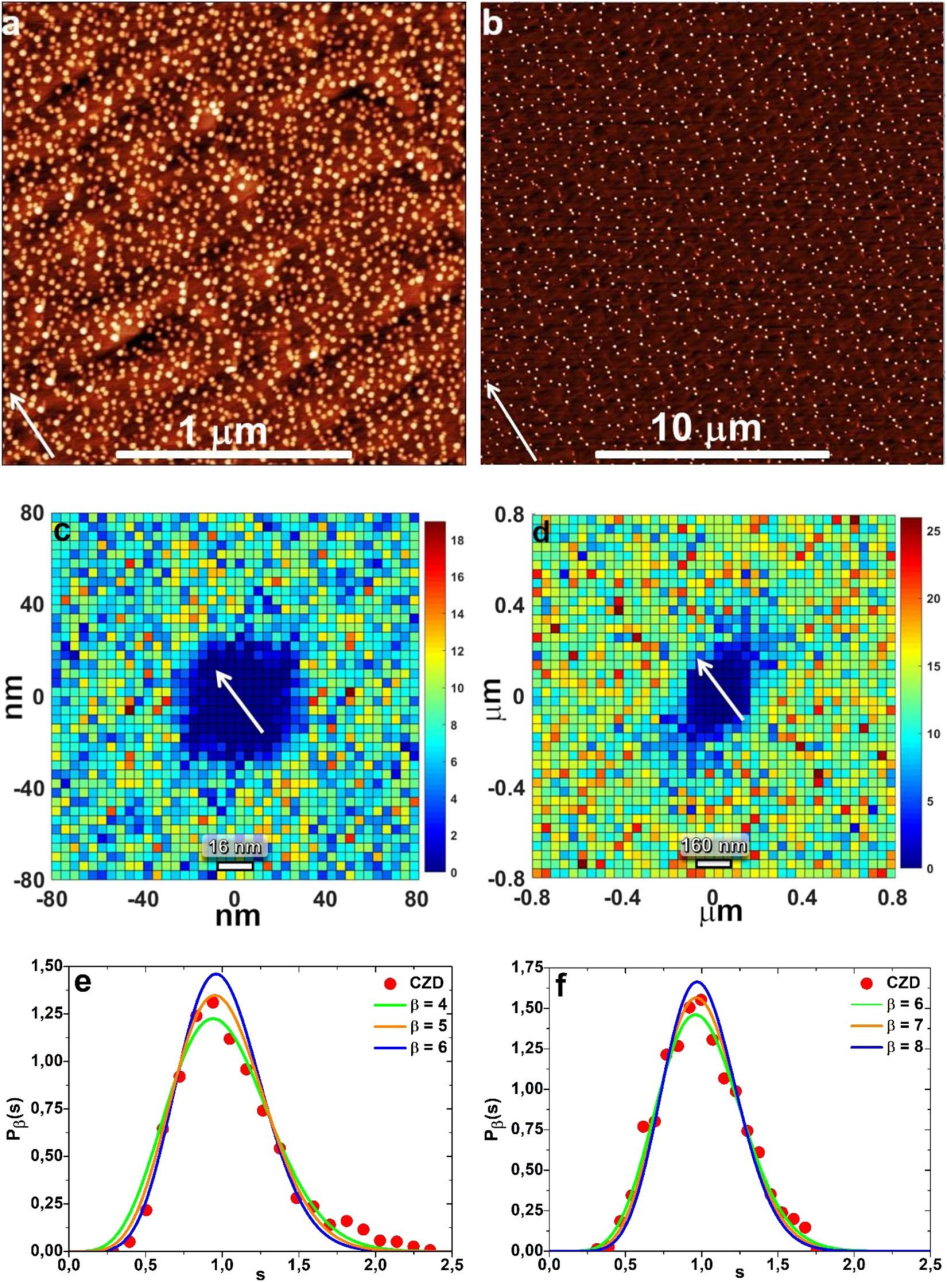
(**a**) AFM image of QDs grown on GaAs(111)A with 2° miscut towards $(\bar{1}\bar{1}2)$ at 300 °C (2 × 2 μm^2^, sample T1), corresponding (**c**) spatial dispersion of neighboring QDs (0.16 × 0.16 μm^2^) and (**e**) CZD obtained from voronoi tessellation, fitted by GWD. (**b**) AFM image of QDs grown on GaAs(111)A with 2° miscut towards $(\bar{1}\bar{1}2)$ at 450 °C (20 × 20 μm^2^, sample T4), corresponding (**d**) spatial dispersion of neighboring QDs (1.6 × 1.6 μm^2^) and (**f**) CZD obtained from voronoi tessellation, fitted by GWD. Arrows indicate $[\bar{1}\bar{1}2]$ direction perpendicular to steps.

Any change in the diffusivity behavior of the adatoms can be monitored through the spatial nearest neighbor distribution of droplets (see Fig. 6c,d). From these graphs it is possible to extract the average excluded zone around each droplet, which is due to the adatom density depletion stemming from the efficient adatom capture by the droplet within one diffusion length from the droplet itself. This makes the adatom density low enough to inhibit additional droplet nucleation in the area^10^. The excluded zone size and shape is then related to the actual adatom diffusion on the surface, and it permits to extract a qualitative dependence of this quantity at different deposition conditions. At *T* < 400 °C, the excluded area has a symmetrical, nearly hexagonal, shape (see Fig. 6c), which show no preferential direction for Ga adatom diffusion. The excluded zone radius is around 20 nm at *T* = 300 °C. This value corresponds approximately to two times the average terrace width. From our AFM scans, we have observed that the droplets nucleate at the terrace steps, thus being able to collect adatoms from two contiguous terraces. The observed excluded zone radius thus stands for to a situation where the adatom, on average, can travel to the droplet without meeting a step. A clear transition in the shape of the excluded zone occurs around 400 °C. At *T* > 400 °C the excluded zone area becomes strongly asymmetrical, with a dimension along $[\bar{1}\bar{1}2]\,\,$exceeding 60 nm and strongly elongated in the $[1\bar{1}0]\,\,$direction, thus along steps. This behavior stems from the presence of a sizeable ES barrier at the step edge which hinders adatom diffusion across the steps^11^. In fact, the ES barrier corresponds to an additional energy needed for an adatom to jump across the terraces^23^, which characterize the (111)A vicinal surface, and decreases the diffusivity in the $[1\bar{1}0]$ direction. Therefore, Ga adatoms at T > 400 °C predominantly migrate in the direction along steps in a strongly anisotropic way, this way approaching a quasi-one-dimensional diffusion behavior.

A second, more accurate, estimation of the critical cluster size *i* can be gained from the analysis of the capture zone (CZ) distribution^24^ of the droplets. CZs can be determined from Voronoi cells, a particular case of surface tessellation where, given a set of centers (in our case the droplets), the space is divided according to their “area of influence”^25^. The CZs derived from the AFM images of the self-assembled droplet at 300 and 450 °C are reported in Fig. 6e,f, respectively. The predicted analytical form of CZ distribution, that is the normalized area distribution of the Voronoi cells, coincides with generalized Wigner distribution (GWD), proposed by Wigner for the nearest-neighbor spacing distribution^21,24,26^:

6 $${P}_{\beta }(s)={a}_{\beta }\cdot {s}^{\beta }\cdot {e}^{-{b}_{\beta }{s}^{2}}$$

where *s* is the CZ area divided by its average value, and ${{\rm{a}}}_{{\rm{\beta }}}=2\Gamma {(\frac{{\rm{\beta }}+2}{2})}^{{\rm{\beta }}+1}/\Gamma {(\frac{{\rm{\beta }}+1}{2})}^{{\rm{\beta }}+2}$and ${b}_{{\rm{\beta }}}={[\Gamma (\frac{{\rm{\beta }}+2}{2})/\Gamma (\frac{{\rm{\beta }}+1}{2})]}^{2}$ are constants that assure normalization and unit mean, respectively, of *P*_*β*_*(s)*^19^. Here $\Gamma (x)$ is the gamma distribution function.

The parameter β is related to the critical nucleus size *i* and to dimensionality of diffusion γ by

7 $$\beta =\gamma i+\gamma +1$$

The experimental normalized capture zone distribution has been obtained from CZ tessellations and have been fitted by generalized Wigner distribution (Fig. 6e,f). On the Fig. 6e, there is a small shoulder at a high capture zone size. This shoulder we attributed to local fluctuations of the terrace sizes or any defects, resulting in a bimodal distribution of capture zones. The fitted β equals 5 ± 1 at 300 and 350 °C while β = 7 ± 1 at 400, 450, and 500 °C. Using Eq. (7), the measured β values correspond, taking into account the two-dimensional adatom diffusion (γ = 1), to critical nucleus size *i* = 3 ± 1 at *T* < 400 °C. At *T* ≥ 400 °C, we observe a quasi-one-dimensional adatom diffusion behavior, so we fit with γ ≈ 2. Then observed β value is consistent with *i* = 2.0 ± 0.5. Therefore, according to GWD analysis, the critical cluster size *i* can be assumed the same in the whole measured *T* range and equal to *i* = 2. This means that three Ga atoms are sufficient to form minimal stable cluster on the vicinal GaAs(111)A surface. The same size for stable Ga cluster was previously demonstrated by *in-situ* scanning tunneling microscopy (STM) during the growth on GaAs(001) substrate^27^. It is worth noticing that the *i* value, as determined by CZ analysis, qualitatively accounts for the observed values of *p*. From Eq. (5) the predicted scaling parameter for one dimensional diffusion at high *T, calculated using i* = 2.0 ± 0.5 and γ = 2, *p*_*HT*_ = 0.29 ± 0.11, and the ratio between the low *T* and high *T* values of *p*, *p*_*HT*_*/p*_*LT*_ = 1.72 ± 0.65, determined setting *i* = 2 and varying γ between its high and low T values, are within the errors of the experimental *p*_*HT*_ = 0.37 ± 0.04 and *p*_*HT*_*/p*_*LT*_ = 2.57 ± 0.52.

## Conclusions

In conclusion, the presence of a sequence of parallel steps and terraces which characterizes the surface of vicinal GaAs(111)A substrates strongly affect the nucleation process of the Ga droplets, formed as precursors of GaAs QDs in DE growth procedure. A crossover between two different diffusion regimes with different dimensionality, occurs at *T* = 400 °C in case of 2° miscut. Two-dimensional isotropic diffusion characterizes the low *T* regime. At *T* > 400 °C, the diffusion becomes highly anisotropic, elongated in the $[1\bar{1}0]$ direction, thus showing that diffusion is hindered by the presence of steps and mainly happens along the terraces. The anisotropy in the adatom diffusivity is the outcome of a sizeable ES barrier, which increases the energy barrier of diffusion across the steps.

This crossover has large effects on the droplet density dependence on *T* and *F*. The critical parameter *p* and activation energy *E*_*a*_ undergo to a reduction of a factor two crossing the critical temperature of the crossover. This means a mild dependence of *N* on the actual growth parameter, thus limiting the droplet density, and the DE-QD engineering opportunities.

CZ distribution analysis, which permits to extract the critical droplet size, shows that *i* equals 2, meaning three Ga atoms are sufficient to form a stable nucleus. This observation appears to be independent on substrate orientation and diffusion dimensionality.
